# Biochemistry and adaptive colouration of an exceptionally preserved juvenile fossil sea turtle

**DOI:** 10.1038/s41598-017-13187-5

**Published:** 2017-10-17

**Authors:** Johan Lindgren, Takeo Kuriyama, Henrik Madsen, Peter Sjövall, Wenxia Zheng, Per Uvdal, Anders Engdahl, Alison E. Moyer, Johan A. Gren, Naoki Kamezaki, Shintaro Ueno, Mary H. Schweitzer

**Affiliations:** 1 Department of Geology, Lund University, 223 62 Lund, Sweden; 2 Institute of Natural and Environmental Sciences, University of Hyogo, 669 3842 Hyogo, Japan; 3 Wildlife Management Research Center, 669 3842 Hyogo, Japan; 4 Mo-clay Museum, 7900 Nykøbing Mors, Denmark; 5 RISE Research Institutes of Sweden, Chemistry and Materials, 501 15 Borås, Sweden; 6 Department of Biological Sciences, North Carolina State University, Raleigh, NC 27695 USA; 7 North Carolina Museum of Natural Sciences, Raleigh, NC 27601 USA; 8 Chemical Physics, Department of Chemistry, Lund University, 221 00 Lund, Sweden; 9 MAX-IV laboratory, Lund University, 221 00 Lund, Sweden; 10 Department of Biosphere-Geosphere Science, Okayama University of Science, 700 005 Okayama, Japan; 11 Department of Ecosystem Studies, University of Tokyo, 113 8657 Tokyo, Japan

## Abstract

The holotype (MHM-K2) of the Eocene cheloniine *Tasbacka danica* is arguably one of the best preserved juvenile fossil sea turtles on record. Notwithstanding compactional flattening, the specimen is virtually intact, comprising a fully articulated skeleton exposed in dorsal view. MHM-K2 also preserves, with great fidelity, soft tissue traces visible as a sharply delineated carbon film around the bones and marginal scutes along the edge of the carapace. Here we show that the extraordinary preservation of the type of *T*. *danica* goes beyond gross morphology to include ultrastructural details and labile molecular components of the once-living animal. Haemoglobin-derived compounds, eumelanic pigments and proteinaceous materials retaining the immunological characteristics of sauropsid-specific β-keratin and tropomyosin were detected in tissues containing remnant melanosomes and decayed keratin plates. The preserved organics represent condensed remains of the cornified epidermis and, likely also, deeper anatomical features, and provide direct chemical evidence that adaptive melanism – a biological means used by extant sea turtle hatchlings to elevate metabolic and growth rates – had evolved 54 million years ago.

## Introduction

**Figure 1:**
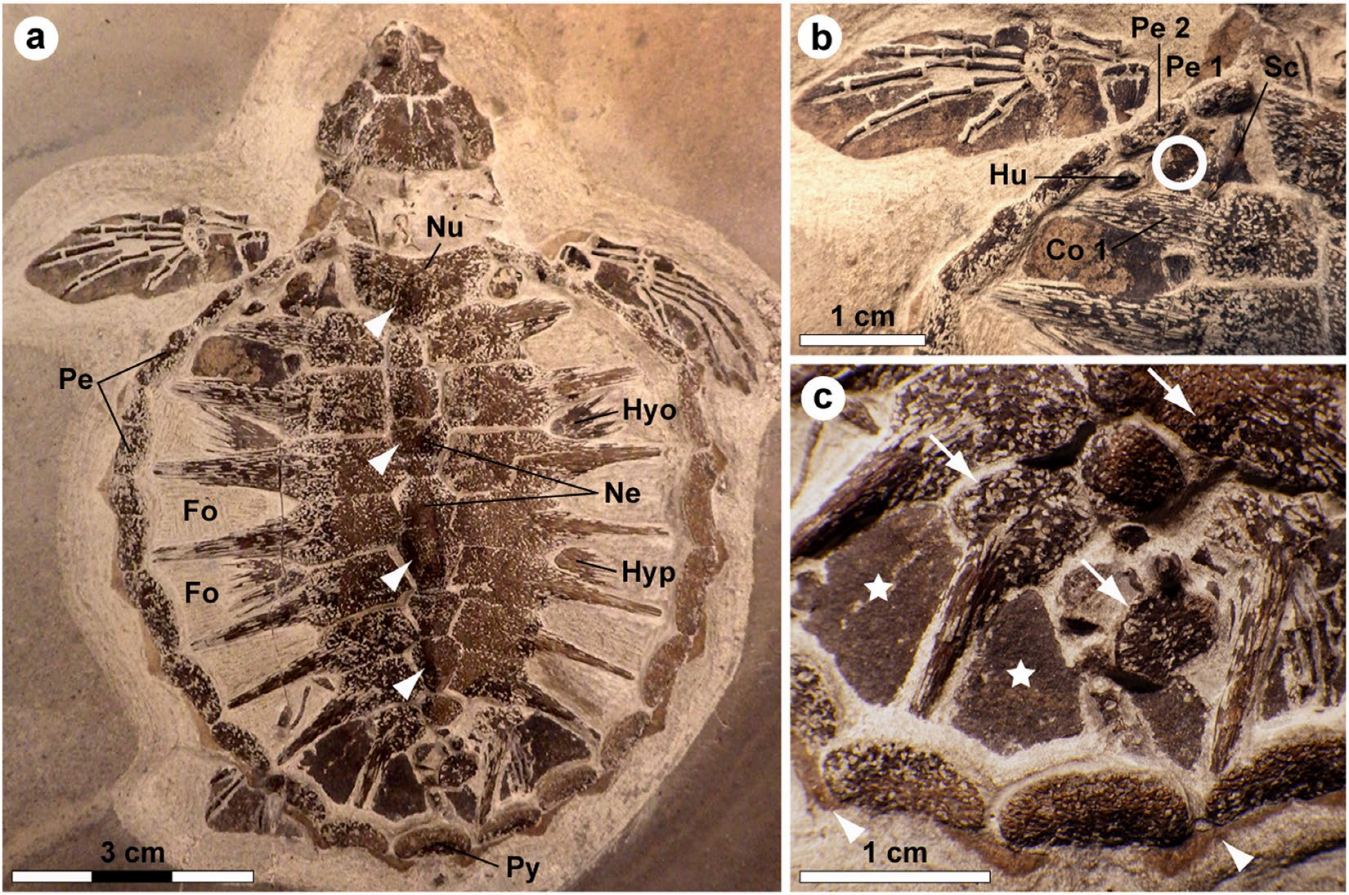
Holotype of *Tasbacka danica*. (**a**) Photograph of the fossil. Fo, fontanelle (the light colour is a result of sediment infill); Hyo, hyoplastron; Hyp, hypoplastron; Ne, neural; Nu, nuchal; Pe, peripheral; Py, pygal. Arrowheads indicate neural nodes. (**b**) Detail of the carapace with the sampled area demarcated by a circle. Co, costal; Hu, humerus; Sc, scapula. (**c**) Higher magnification image showing marginal scutes (arrowheads), pigmentations on bones (arrows), and a brown-black film covering the fontanelles (stars).

Marine deposits of the early Eocene Fur Formation (Jutland, Denmark) have yielded a diverse biota of exceptionally preserved plant and animal fossils^1^. Noteworthy finds include a speciose assemblage of early neornithes^2^ and a small, but significant, collection of chelonioid turtles^3,4^. Of the latter group, by far the best preserved specimen is MHM-K2: a diminutive (about 74 mm when measured from the nuchal emargination to the posterior rim of the pygal), ontogenetically young cheloniine referred to as *Tasbacka danica* by Karl & Madsen^5^. MHM-K2 was collected in 2008 from within a limestone concretion in the Ejerslev Mo-clay pit on the Isle of Mors, Denmark, by one of us (HM). Initially, the fossil was prepared using a combination of mechanical tools and buffered acetic acid. However, following the discovery of soft tissue remains, only a pneumatic air scribe was employed to expose the dorsal aspect of a virtually intact turtle skeleton showing true bone-to-bone relationships, save for the eighth right costal and suprapygal (Fig. 1a). Although the fossil has suffered some compaction damage affecting parts of the skull, the individual bones are generally in pristine condition and retain their original three-dimensional shape. The carapace has a broadly ovate outline with a concave anterior margin, and it is about as wide as it is long in its current, compressed state (Fig. 1a). Large spaces (fontanelles) occur between the ribs and peripherals, and sculpturing in the form of four neural nodes can be seen along its antero-posterior midline (Fig. 1a—arrowheads). The plastron is mostly obscured by the carapace; however, parts of the lateral processes of both the hyo- and hypoplastron are visible in the fontanelles enclosed by the second and third, and fourth and fifth costal, respectively. Adaptations for an obligate marine existence (e.g. elongate phalanges) are apparent in the paddle-shaped limbs (Fig. 1a,b); notably though, the extremities are proportionally short by sea turtle standards. Vestiges of soft tissues include buff-coloured marginal scutes lining the outside of the peripherals (Fig. 1a,c—arrowheads), and dark matter forming well-defined outlines of the neck and flippers (Fig. 1a,b), as well as covering a number of bones and fontanelles (Fig. 1c—arrows and stars).

Here we examine, at the molecular level, details of the soft tissue anatomy of MHM-K2. Renewed preparations of the fossil in 2013 uncovered soft tissue residues from a ‘fresh’ sub-surface layer located within a sub-triangular area bordered by the dorsal process of the left scapula, proximal end of the left humerus, left costal 1, and left peripherals 1 and 2 (Fig. 1b). Five small samples representing the counterpart of MHM-K2 were collected for ultrastructural and biomolecular analysis; these were untreated with preservatives and form the basis of this study (the fossil has since been embedded in consolidants while making replica casts, thereby precluding the recovery of additional samples for molecular analysis).

To determine the ultrastructural and chemical composition of the preserved soft tissues, both untreated and demineralised (using ethylenediaminetetraacetic acid, EDTA) samples were subjected to a selection of high-resolution analytical techniques, including field emission gun scanning electron microscopy (FEG-SEM), transmission electron microscopy (TEM), *in situ* immunohistochemistry, time-of-flight secondary ion mass spectrometry (ToF-SIMS), and infrared (IR) microspectroscopy.

## Results

### Ultrastructural and elemental analyses

**Figure 2:**
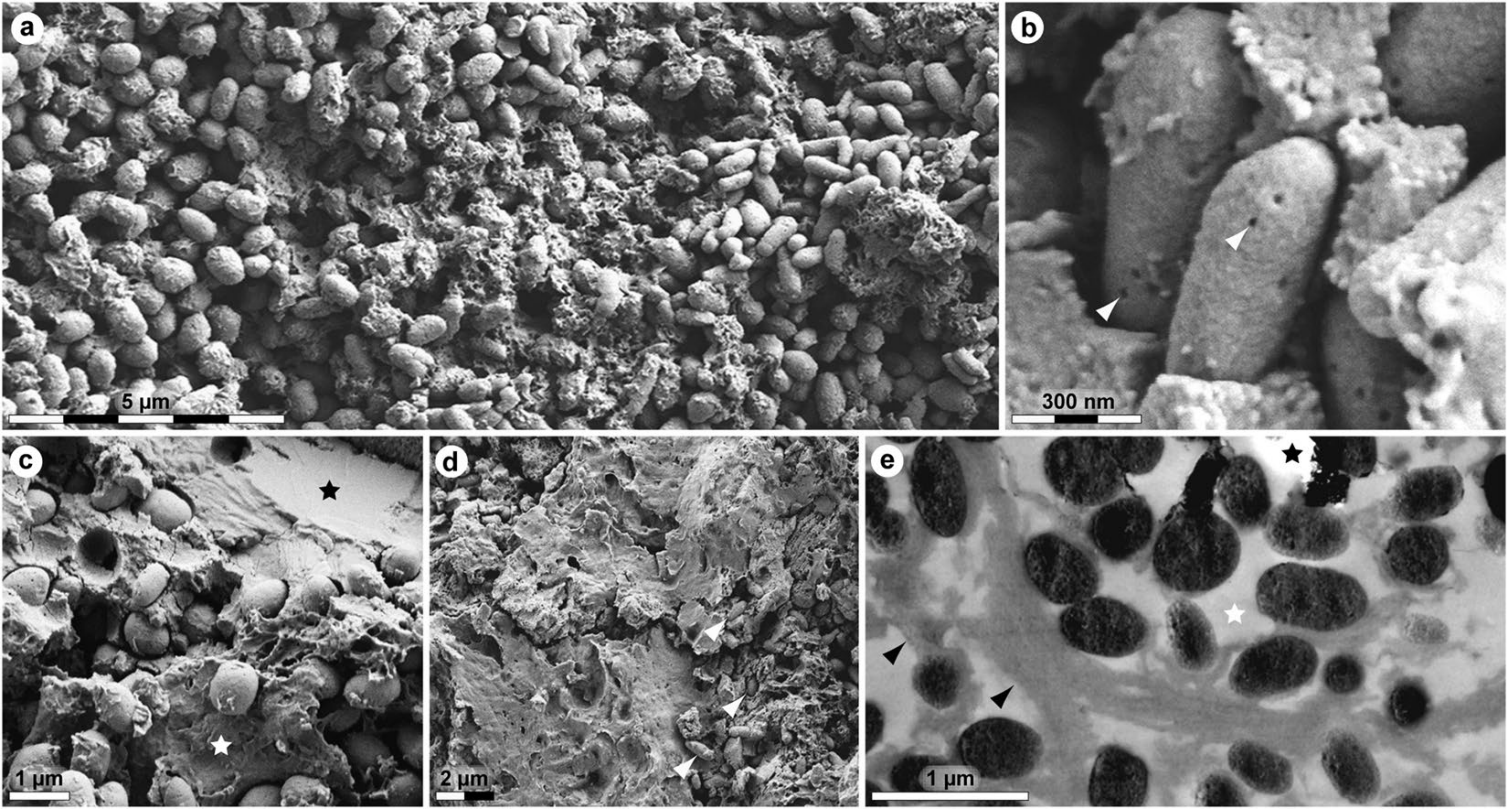
Ultrastructure of MHM-K2 soft tissues. (**a**) FEG-SEM micrograph of demineralised tissue showing microbodies and adhering matrix. (**b**) At higher magnification, the microbodies possess a rough surface texture and scattered pits (arrowheads). (**c**) FEG-SEM micrograph of untreated soft tissue depicting microbodies embedded in a mineral precipitate (black star) and sheet-like matter (white star). (**d**) Microbodies (arrowheads) in a sheet-like substrate. (**e**) TEM micrograph of electron-dense microbodies and fibrous matrix (black arrowheads) after demineralisation. White star indicates epoxy resin, whereas black star marks an artificial rupture.

Initial macroscopic examination of the preserved soft tissues showed a dark, well-defined film that is distinct from the surrounding sediment in both texture and colour. Subsequent FEG-SEM analysis revealed that the thin layer comprises accumulations of sub-spherical to elongate microbodies with a homogenous interior and botryoidal, irregularly pitted exterior (Fig. 2a–c). These are encapsulated within a matrix consisting of two main types of fabric: a fine-grained material interpreted as an authigenic precipitation (Fig. 2c—black star), and, more commonly, a three-dimensional meshwork with a sheet-like to vesicular (frothy) texture (Fig. 2c—white star and Fig. 2d). Elemental analysis using energy-dispersive X-ray microspectroscopy established that the microbodies and sheet-like substrate are enriched in carbon, indicating the possible retention of organic compounds.

TEM imaging revealed that the film, although only 15 µm in depth, has a three-dimensional configuration. Furthermore, the electron-dense microbodies are neither stacked nor overlapping, but rather separated by a fibrous matrix that defines the interstitial spaces (Fig. 2e—arrowheads).

### Immunohistochemical analyses

**Figure 3:**
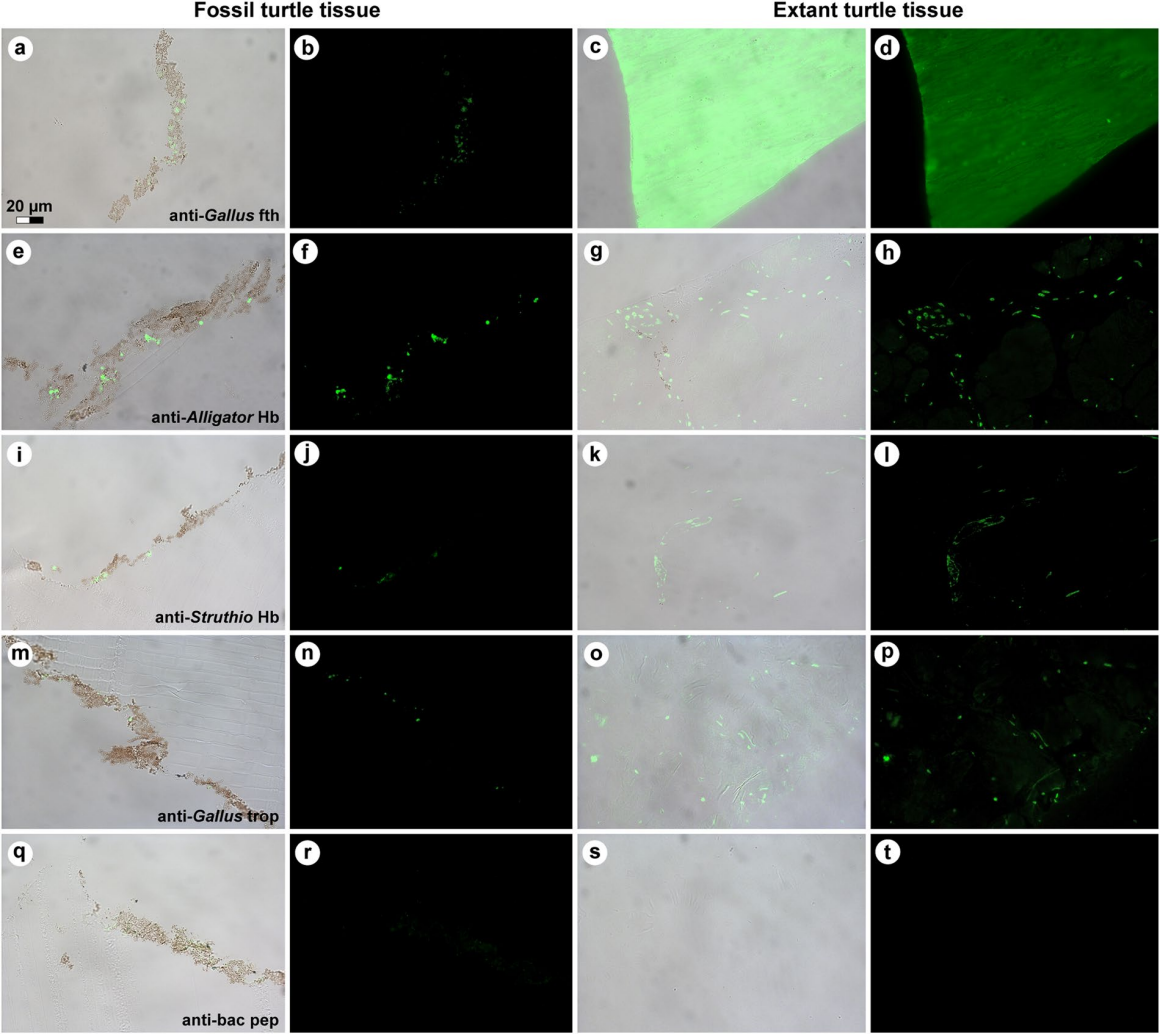
Immunoreactivity of fossil and extant turtle tissues. Immunohistochemical staining results for (**a**,**b**,**e**,**f**,**i**,**j**,**m**,**n**,**q**,**r**; columns 1 and 2) MHM-K2 and *Chelonia mydas* (**c**,**d**; columns 3 and 4) carapace scute and (**g**,**h**,**k**,**l**,**o**,**p**,**s**,**t**; columns 3 and 4) muscle tissue to antibodies raised against (**a**–**d**; row 1) *Gallus gallus domesticus* feathers (anti-*Gallus* fth), (**e**–**h**; row 2) *Alligator mississippiensis* haemoglobin (anti-*Alligator* Hb), (**i**–**l**; row 3) *Struthio camelus* haemoglobin (anti-*Struthio* Hb), (**m**–**p**; row 4) *G*. *g*. *domesticus* tropomyosin (anti-*Gallus* trop), and (**q**–**t**; row 5) bacterial peptidoglycan (anti-bac pep). **a**,**c**,**e**,**g**,**i**,**k**,**m**,**o**,**q**,**s** are overlay images, superimposing fluorescent signal on transmitted light image of sectioned tissue to reveal the localisation of antibody-antigen complexes to tissue. **b**,**d**,**f**,**h**,**j**,**l**,**n**,**p**,**r**,**t** are imaged using a FITC filter. Antibody-antigen complexes are indicated by green fluorescent signal.

**Figure 4:**
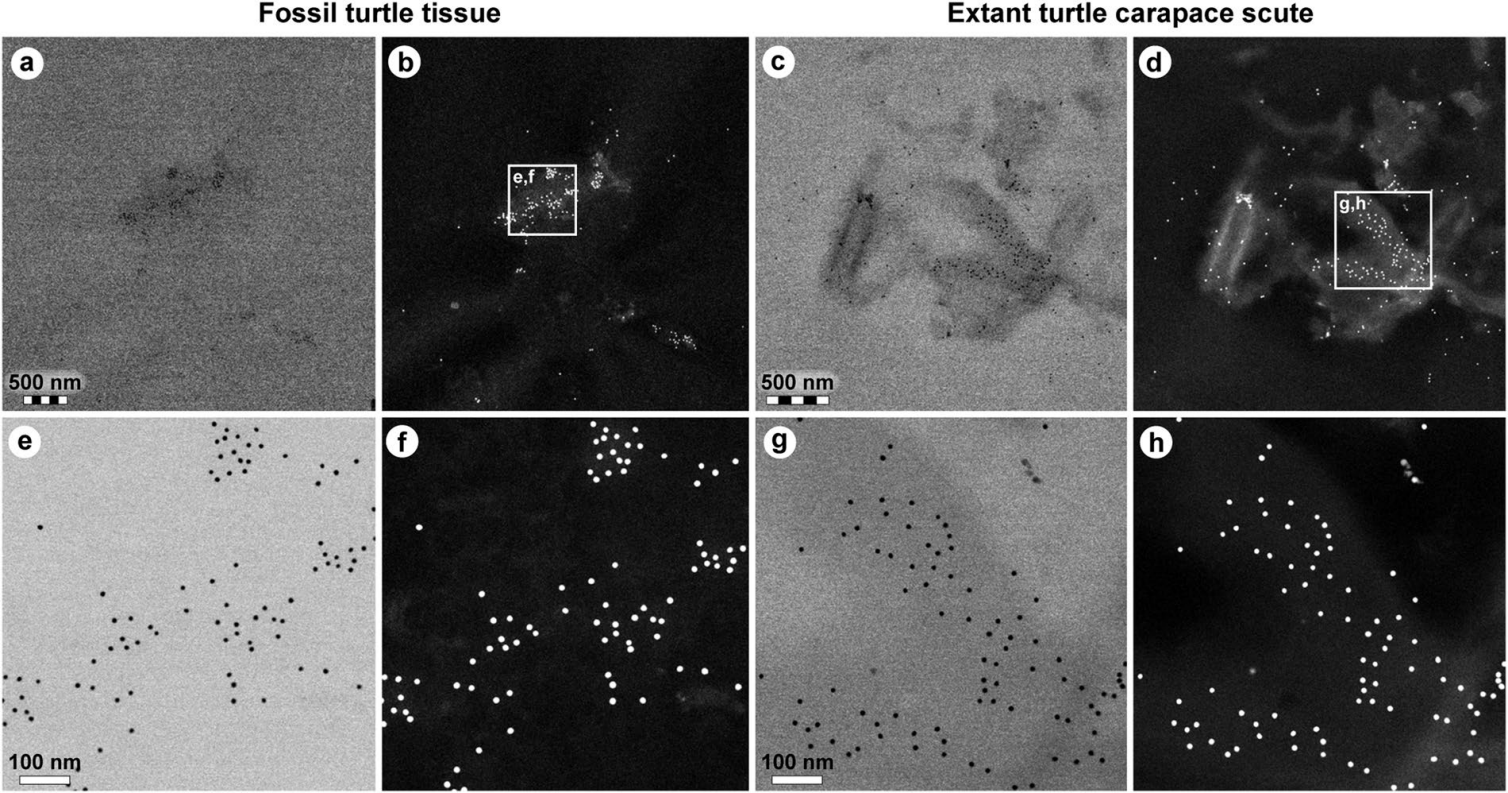
Comparison of immunoreactivity between fossil and extant turtle tissues using antibodies raised against chicken feathers conjugated to 12 nm gold beads. (**a**–**d**) Low and (**e**–**h**) high resolution localisation of gold beads to fibrous matter, but not microbodies/melanosomes, in (**a**,**b**,**e**,**f**) fossil tissues and (**c**,**d**,**g**,**h**) modern *Chelonia mydas* carapace scute material. Insets in **b** and **d** demarcate areas depicted in **e**,**f** and **g**,**h**, respectively. The data support the specificity of the chicken feather antibodies used in this study, and provide independent validation of the immunofluorescent results.

In living chelonians, the bony carapace is covered by an epidermis comprised mainly of keratinous proteins. Both α- and β-keratins are present; however, the latter is only expressed in sauropsid cornified tissues^6,7^, and thus cannot arise from human or microbial contamination. Accordingly, we employed the specificity of the vertebrate immune system (Figs 3, 4 and Supplementary Fig. S1) to distinguish between endogenous animal remains and invasive microorganisms that naturally colonise decaying organic matter^8–10^. We used antibodies raised against chemically extracted white feathers (which almost exclusively comprise β-keratin) from chicken, *Gallus gallus domesticus* ^9,11^, to test the hypothesis that the structurally distinct brown-black residue associated with the bony shield of MHM-K2 was consistent with β-keratin of the epidermal coating of the carapace^12–14^. Regions of this protein are homologous in turtles and birds^7,12,13,15^; therefore, if original molecules (or remains thereof) were preserved, we predicted that they would be recognised by these antibodies. Antibody-antigen complexes were identified by green fluorescence *in situ* on the ancient matter (Fig. 3a,b), and compared to the pattern observed in scute material obtained from an extant green sea turtle, *Chelonia mydas*, using the same antibodies and experimental parameters (Fig. 3c,d and Supplementary Fig. S1). Although the binding was less intense and more punctate in MHM-K2 relative to its modern counterpart, it was well above background levels, indicating that epitopes (that is, three to five amino acid residues derived from the protein and retaining their original three-dimensional conformation) remained in the fossil tissues. Furthermore, these antibodies bound to the ancient matter with the same pattern as that observed in comparable extant tissues, and were restricted to areas in between the fossil microbodies (Fig. 3a).

To obtain higher resolution of the antibody-antigen complexes, binding was also detected using a secondary antibody conjugated to 12 nm gold beads. Specific localisation of beads to the filamentous matrix was evident in both the fossil (Fig. 4a,b,e,f) and modern (Fig. 4c,d,g,h) turtle tissues.

To test the hypothesis that structures deeper than the cornified epidermis might be preserved in MHM-K2, we exposed fossil material to antibodies raised against haemoglobin from both the American alligator, *Alligator mississippiensis*, and ostrich, *Struthio camelus*, as well as antibodies raised against chicken tropomyosin (an eukaryotic cytoskeletal protein^16^). Reactivity to antibodies against haemoglobin and tropomyosin was evident in MHM-K2 (Fig. 3e,f,i,j,m,n) in patterns similar to those in *C*. *mydas* muscle tissue (Fig. 3g,h,k,l,o,p), but with reduced intensity.

Finally, to address the possibility that some of the microbodies and/or organic matrix in which they are housed could be microbially derived, we tested the fossil and modern tissues against antibodies raised against peptidoglycan – a common component of bacterial biofilms^17^. Whereas reactivity was absent in the control samples (Fig. 3s,t), weak antibody response was visualised in MHM-K2 (Fig. 3q,r), suggesting a minor (but not unexpected) microbial contribution.

### Time-of-flight secondary ion mass spectrometric analyses

**Figure 5:**
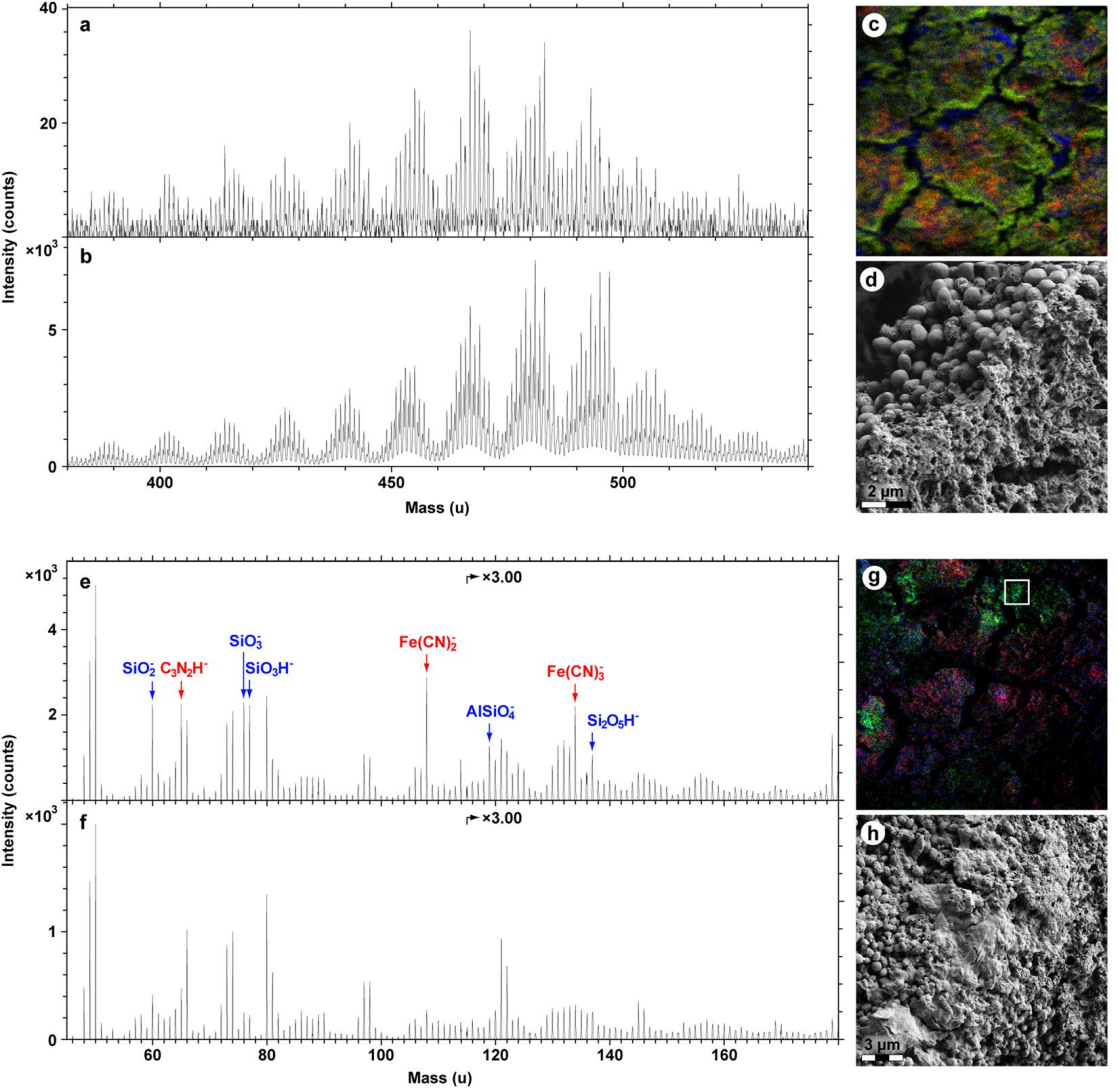
Molecular characterisation of MHM-K2 tissues by ToF-SIMS analysis. (**a**) Positive ion spectrum from a region with strong signal from heme-related ions. (**b**) Positive ion spectrum of a heme (hemin) standard. (**c**) Negative ion image showing the signal intensity distribution of ions representing heme (red; 65 + 108 + 134 u), eumelanin (green; 66 + 73 + 74 + 97 + 98 + 121 + 122 u) and silica (blue; 60 + 76 + 77 u). Field of view: 200 × 200 µm^2^. (**d**) FEG-SEM micrograph showing a fracture edge. Note abundant microbodies in the crack wall and vesicular texture of the surface. (**e**) Negative ion spectrum from a region with mixed signal from eumelanin- and heme-related ions. (**f**) Negative ion spectrum from a region dominated by signal from eumelanin-related ions. (**g**) Positive ion image showing the signal intensity distribution of ions representing heme (red; 436–488 u), aromatics (blue; 91 + 115 u) and proteinaceous materials (green; 30 + 44 + 70 u). Field of view: 328 × 328 µm^2^. (**h**) FEG-SEM micrograph of the demarcated area in **g** depicting sheet-like matter with high signal from amino acid-related peaks.

**Figure 6:**
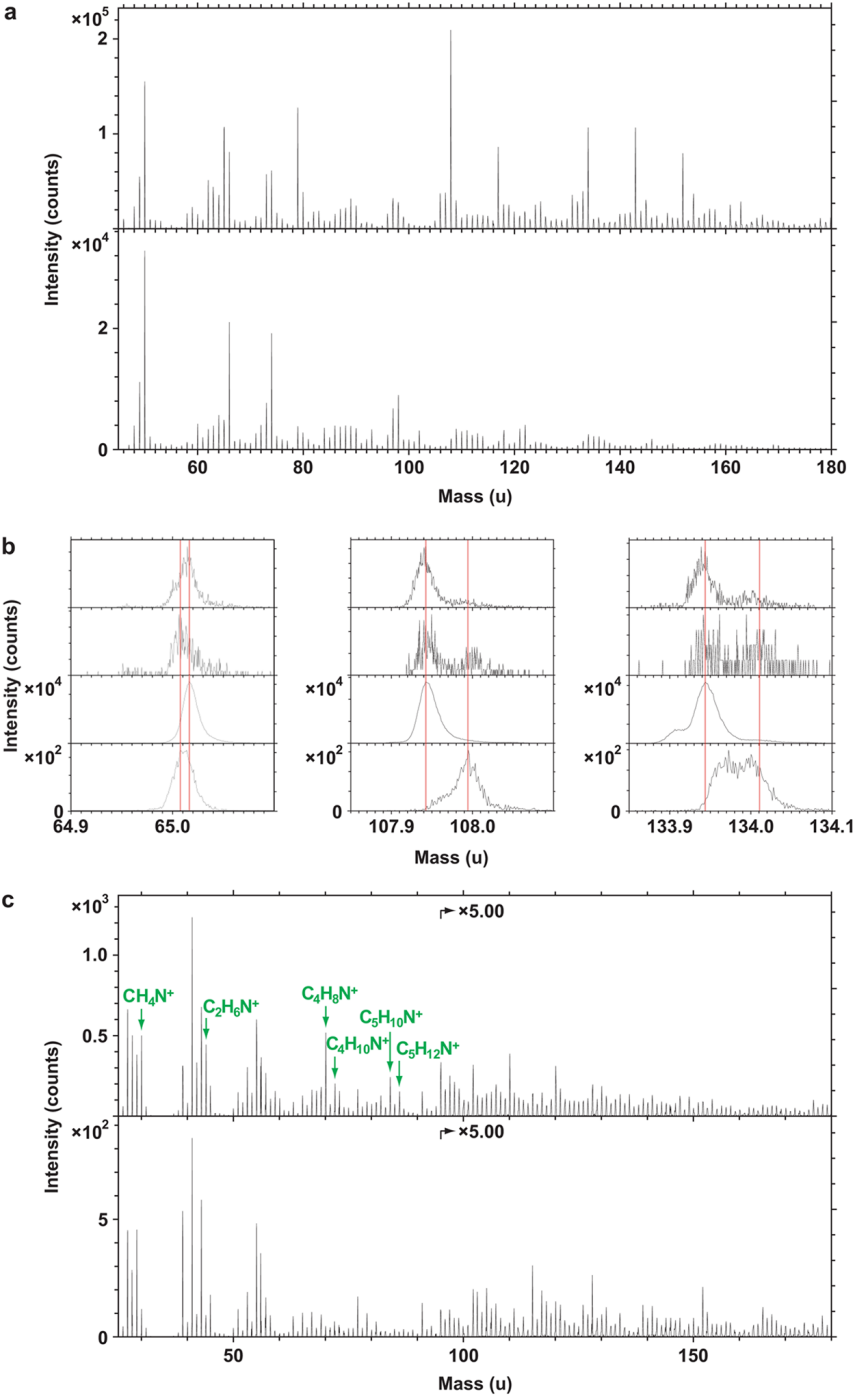
ToF-SIMS spectra obtained from MHM-K2 soft tissues together with selected standards. (**a**) Negative ion spectra of hemin (top) and *Sepia* eumelanin (bottom) standard compounds. (**b**) Expanded negative ion spectra of (from top to bottom): MHM-K2, from an area dominated by eumelanin-related ions; MHM-K2, from an area with mixed signal from eumelanin- and heme-related ions; *Sepia* eumelanin, and; hemin. The depicted masses (65, 108 and 134 u) all show strong signal in the hemin reference spectrum. (**c**) Positive ion spectra acquired from regions of MHM-K2 with strong signal from amino acid- (top) and heme-related (bottom) ions, respectively. Peaks marked in green represent ions characteristic of proteinaceous materials.

ToF-SIMS data obtained from the fossil soft tissues provided evidence for the presence of heme, eumelanin and proteinaceous matter (Figs 5 and 6), thereby independently validating the immunological results.

Heme was identified in positive ion mode as a distinct set of undulating peaks in the mass range between 400 and 500 u (Fig. 5a); this fragmentation pattern was reproduced in detail in spectra acquired from a purified heme control sample (Fig. 5b). The peaks derive from ions comprising the entire iron-containing porphyrin unit of heme after the successive loss of various CH_x_ entities^18,19^. However, the molecular ion (that is, the intact heme molecule in its ionised form) was not detected in the fossil spectra, indicating partial, likely diagenetically mediated degradation of the heme molecular structure (including loss of various -COOH units^18^). Compounding this identification, heme-derived molecular fragments were observed in negative ion spectra e.g. at 65 (C_3_N_2_H^−^), 108 [Fe(CN)_2_^−^] and 134 u [Fe(CN)_3_^−^]; these peaks occurred at high signal intensities also in the heme standard spectra (Fig. 6a,b).

The identification of eumelanin on the fossil surfaces was based on its detailed spectral agreement in the mass range between 45 and 175 u (negative ions) with a *Sepia* eumelanin standard sample (Figs 5f and 6a,b), both with regard to the relative signal intensity distribution and exact mass positions^10,20–22^.

In addition to heme and eumelanin, a third compound showing spectral features typical of proteinaceous materials (such as strong signal intensity from specific nitrogen-containing ions representing amino acids^23^) was also recognised (Fig. 6c). Notably, the majority of the amino acid-related peaks were not prominent in ToF-SIMS spectra of either eumelanin or heme, to suggest a different molecular origin of these ions.

The eumelanin- and heme-related ions showed relatively homogeneous distributions on the fossil surfaces, but with some noteworthy differences. Particularly, areas deficient in heme, but with strong signal from eumelanin-related peaks, could be found at edges of desiccation cracks running across the sample surfaces (Fig. 5c). Subsequent FEG-SEM analysis revealed the occurrence of densely packed microbodies in association with a vesicular film; the latter originally occurring medial to the microbody-rich layer (Fig. 5d). Negative ion spectra taken from regions comprising a mixture of bodies and vesicular matter showed characteristics of both eumelanin and heme (Figs 5e and 6a,b), whereas areas dominated by microbodies produced spectra typical of eumelanins (Figs 5f and 6a,b). Regions with strong signal intensity from amino acid-related peaks were relatively scarce and their distribution was distinct from that of both heme and eumelanin, to suggest the presence of scattered proteinaceous residues on the fossil surfaces (Figs 5g and 6c). FEG-SEM imaging of areas with amino acid signal showed sheet-like material (Fig. 5h), interpreted as partially degraded keratin plates.

### IR microspectroscopic analyses

**Figure 7:**
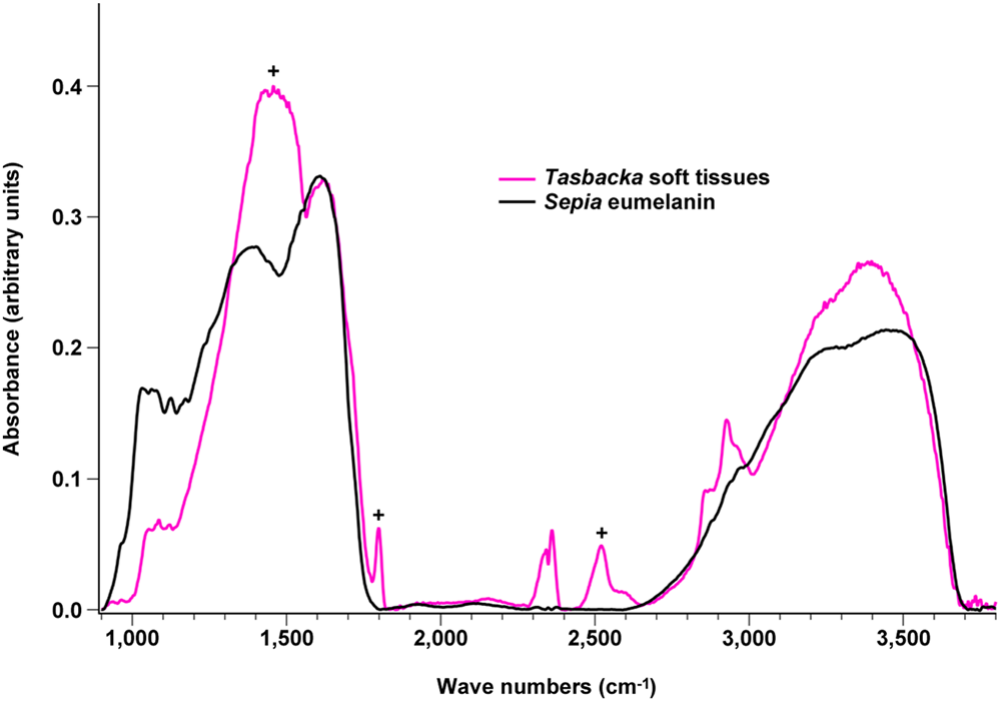
IR absorbance spectra of *Tasbacka* soft tissues and *Sepia* eumelanin. The *Tasbacka* spectrum exhibits broad-band absorbance in the 900–1,750 and 2,700–3,700 cm^−1^ regions, consistent with the natural eumelanin standard. Differences (marked with ‘+’) can be attributed to contributions from the sedimentary matrix (mainly calcite)^20,68^. These occur also in previously analysed samples from the Fur Formation^20^. For example, the intense and broad peak at ~1,450 cm^−1^ likely includes absorbance from the asymmetric stretch mode of calcium carbonate^68^.

IR microspectroscopic measurements of the fossil tissues produced broad-band absorbance in the 900–1,750 and 2,700–3,700 cm^−1^ regions, consistent with natural eumelanin (Fig. 7).

## Discussion

The methods employed herein were chosen to obtain maximal information while minimising sample destruction. They are highly specific and extremely sensitive, yet capitalise on different aspects of the molecules they characterise. Accordingly, the results of our integrated ultrastructural and biochemical approach provide compelling independent lines of evidence for the presence of an unprecedented number of endogenous biomolecular residues, including haemoglobin-, β-keratin-, tropomyosin-, and melanin-derived compounds (as well as residual peptidoglycan), in the soft tissue remains recovered from MHM-K2. Moreover, the anatomical localisation, geometry and size of the fossil microbodies and adhering matrix compare favourably with those of remnant melanosomes (Fig. 2c in ref.^21^) and degraded keratin plates (Supplementary Fig. S2; see also Fig. 10c–e in ref.^14^), respectively, to suggest that the preserved organic matter comprises vestiges of the pigmented keratinous epidermal coating of the shell. This interpretation is supported not only by the presence of β-keratin epitopes in the fibrous substrate surrounding the microbodies (Figs 3a,b and 4a,b,e,f), but also by their electron-dense interior (Fig. 2e) – a distinguishing feature of mature melanosomes^10^. Additionally, marginal scutes are visible alongside the peripherals (Fig. 1a,c—arrowheads), and stains on some bones indicate that the soft tissues are located outside of the ribcage (Fig. 1c—arrows). Incomplete decomposition of the carapace due to oxygen deprivation and/or early sealing of the entombing sediment by concretion formation^2,24^ (perhaps facilitated by microbial mat growth^25^) presumably reduced the keratin laminate (that is, the stacked layers of cornified cells and β-pleated sheet keratin forming the scutes^14,26^), thereby concentrating the more decay-resistant (refs^27,28^, but see also ref.^9^) melanosomes into a condensed layer, which in turn underwent compactional flattening. Preferential preservation of integumental features may seem unlikely; however, skin-derived structures are second only to biomineralised tissues in the vertebrate fossil record (ref.^29^) and references therein), and decay experiments on modern carcasses have revealed the persistence of integumental tissues even after long-term exposure to microbial decomposers^25,30^. In the Fur Formation, carbonised animal soft tissues, with the notable exception of orbital (retinal) pigmentations^10,20^, chiefly comprise integumental features (often with preserved colour patterns; Figs 38, 39a and 46 in ref.^31^, Fig. 1 in ref.^32^).

In extant vertebrates, skin forms a tough physico-chemical barrier that performs multiple functions^33^. Additionally, turtle scutes constitute a resilient shielding material which provides the shell with a compliant coating that enhances its resistance to mechanical damage^26,34^. Recalcitrant biopolymers, including keratins and melanins, are key components of integumental appendages; their hydrophobic nature and inherent ability to form robust intra- and intermolecular cross-links likely contribute to the survival of epidermal structures across geological time^9,22,29,35^. Besides being impermeable, most epidermally-derived features are also avascular^33^, thus reducing the possible entry of degrading microbes.

Another factor contributing to the selective preservation of skin and its derivatives may be the close spatial juxtaposition between these anatomies and the host rock. Sorption of organics onto clay minerals (a common component of the Fur Formation^36^) promotes fixation^37–39^; clays also possess antibiotic properties^40^. Furthermore, association of biological matter with mineral nano- and microfabrics adds protection from oxidative and hydrolytic damage through the creation of stabile organometallic complexes^41,42^. Chemical evidence for this process is seen in MHM-K2 as a series of molecular fragments involving calcium adducts (Table 1). We hypothesise that calcium ions (and other trace elements) adsorbed onto the surface of the carcass during the microbially mediated^24^ formation of the calcareous concretion in which MHM-K2 was found. Mild geothermal conditions^43^ might then have limited further breakdown of the stabilised organics.

**Table 1:** Organometallic fragments involving calcium adducts.

	Observed mass (u)	Theoretical mass (u)
C_2_HCa^+^	64.973	64.970
CNCa^+^	65.969	65.966
C_2_H_3_Ca^+^	66.987	66.986
COHCa^+^	68.965	68.965
CNOCa^+^	81.958	81.961
C_2_H_3_OCa^+^	82.982	82.981
C_4_HCa^+^	88.971	88.970
C_3_NCa^+^	89.972	89.966
C_3_NOCa^+^	105.952	105.961
C_2_H_3_N_2_OCa^+^	110.978	110.987

Positive ion ToF-SIMS data obtained from MHM-K2 demonstrating the intimate association between the organic residues and mineral substrate.

Haemoglobin also imparts tissue fixation by iron-catalysed free radical reactions and/or inhibition of bacterial growth (ref.^44^ and references therein), possibly contributing to preservation of anatomical features deeper than the cornified epidermis. Blood breakdown products released from erythrocytes during hemolysis can seep into surrounding tissues, causing a reddish-brown discolouration^45^. Impregnation by haemoglobin-derived compounds has been recorded not only in bones^46^, but also in scales and teeth^45^. Consequently, it is possible that the outer integument was infiltrated by blood residues diffusing from underlying (and now almost completely degraded) dermal or deeper tissues sometime during the early stages of decomposition of MHM-K2. Detection of haemoglobin- and tropomyosin-derived compounds supports this possibility.

Extant neonate sea turtles are generally predominantly dark-coloured, at least on their dorsal surface (Supplementary Fig. S3). In most species, this dusky patterning changes with age^47,48^. Thus, its selective advantage is not immediately apparent, especially since the brown-black pigmentation increases the risk of overheating while on land (owing to enhanced absorbance of solar radiation)^47^, but also because it makes the hatchlings stand out against the beach sand during their dash towards the sea (Supplementary Fig. S3a,b). However, potential selective benefits are illustrated by the green sea turtle, *Chelonia mydas*. Hatchlings of this species are dark brown to black dorsally, save for white edgings on the carapace and flippers (Supplementary Fig. S3c). The plastron, on the other hand, is lightly coloured (ref.^48^, but see also ref.^49^). When emerging from the nest, hatchling *C*. *mydas* face the same challenges as do other sea turtles; however, the functional significance of the dark dorsum becomes more readily apparent once in their pelagic habitat. While resting, neonate *C*. *mydas* float at the sea surface with large parts of the body above the waterline (Fig. 1 in ref.^47^). In this posture, the dark upper surface provides ultraviolet (UV) ray protection and camouflage^50^, but also more efficient absorption of solar radiation, thereby promoting an elevation of the body temperature^47^. The resulting rise in basal metabolic rates contributes to increased growth rates during this vulnerable early life stage^47^.

In ectotherms, incident radiation is converted into thermal energy primarily by integumental eumelanins^51^; there is also a direct relationship between the density of these pigments and skin darkness^51–53^. In addition to melanins, non-endothermic vertebrate skins often contain a number of other biochromes, which together with finely organised nanostructures causing light interference, produce the variety of colour patterns seen in fish, amphibians and reptiles today^32,52–54^. However, contrary to recent claims^55^, non-melanic pigments and photonic nanosurfaces are *not* ubiquitous to all reptile skins. For instance, the colour of neonate *C*. *mydas*, *Caretta caretta* (loggerhead sea turtle) and *Dermochelys coriacea* (leatherback sea turtle) is determined exclusively by melanins: dark portions of the integument are defined by abundant melanosomes and melanophores (Supplementary Fig. S4a,b,d,e,g–n), whereas light portions possess few melanophores or lack pigment cells altogether (Supplementary Fig. S4c,f,o). Similar observations have previously been made by Solomon *et al*.^26^, to suggest that contributions from light-reflecting biochromes and materials are negligible in melanised marine reptile skins^21^. Additionally, countershading is consistent with life in the featureless pelagic zone, where there are few hiding places and thus little need for bright colours or disruptive markings^56^.

With a carapace length roughly corresponding to that of four- to five-week old *C*. *mydas* ^49^, MHM-K2 represents an ontogenetically very young individual^5^. Apart from size, skeletal immaturity is manifested by the incomplete development of the costals – resulting in large fontanelles – and presence of neural nodes along the carapace midline (Fig. 1a—arrowheads). These juvenile features, together with the findings of this study, combine to suggest strongly that the type of *Tasbacka danica* originally had a predominantly dark dorsum with pale edges to the carapace (although concentrated by scute reduction, the sheer number of melanosomes in MHM-K2 indicates that the original density was high, equalling that of modern chelonioids). Thus, it was likely similar in appearance to extant sea turtle hatchlings, particularly *C*. *mydas* (Supplementary Fig. S3c), and may even have relied on melanism as an integral part of its thermoregulation. Although we cannot completely exclude the possibility that there may have been biochromes and/or nanostructures that did not survive long enough to enter the fossil record, our interpretation is corroborated by the fact that the colouration of most (if not all) modern neonate chelonioids is produced solely by melanins (Supplementary Fig. S4). We also acknowledge the inherent uncertainty associated with extrapolations of large-scale pigment patterns from minute samples^57^. However, in this case, a partial colour reconstruction is justifiable because: (1) the fossil preserves a distinct patterning incorporating both light (marginal scutes) and dark (fontanelle coverings) areas; (2) a relationship exists between melanosome density and apparent darkness of preserved soft tissues in Fur Formation vertebrate fossils^10,20,21,58,59^; (3) there is a close phylogenetic relationship between *T*. *danica* and living cheloniines^5^; and (4) extant neonate sea turtles exhibit relatively uniform, darkly pigmented carapaces^48,60^. Likewise, we exclude major contributions from dispersed internal melanosomes^61^ because the fossil pigment organelles in MHM-K2 are embedded within remnant β-keratin, consistent with the epidermal covering of the shield. Additionally, blackish soft tissues occur also in the distal end of the flippers (Fig. 1a,b), which are far from any potential melanin-harbouring internal source.

Our results suggest that a biological means of UV protection and/or melanin-based thermoregulation existed in hatchling cheloniines at least 54 million years ago (notably in the pronounced greenhouse climate following the Palaeocene-Eocene Thermal Maximum). However, because melanism is widespread among extant neonate chelonioids and because they share a common ancestor in the Early Cretaceous (or possibly Late Jurassic)^62,63^, phylogenetic bracketing^64^ implies that this adaptation may have appeared already during the latter part of the Mesozoic. If so, then it was secondarily lost in the evolutionary lineage leading to the modern flatback sea turtle, *Natator depressus* (hatchlings of this species have a light grey dorsum; Fig. 1b in ref.^65^), perhaps due to marked size differences at birth^47^ or because neonate *N*. *depressus* employ survival strategies different from those of other marine turtles^65^.

## Methods

### Fossil material

MHM-K2 is housed in the collections at the Mo-clay Museum, Denmark (the holotype of Tasbacka danica has recently been transferred to the Natural History Museum of Denmark. New accession number: NHMD 141598). The fossil tissue samples were triple-washed in 96% ethanol and Milli-Q water to remove potential contaminants from human handling. They were then dried under a hood, wrapped loosely in fresh aluminium foil and stored in a sealed, sterile container. Fresh aluminium foil was used to cover all work areas, and surgical gloves were used during all handling and treatment.

### Modern reference materials

The body of a hatchling green sea turtle, *Chelonia mydas* (KPC16030901; housed in Kamezaki public collection), that had died from natural causes, was collected on the Amami Island of Japan in August of 2011 and has since been stored in a freezer. Similarly, a second (juvenile) specimen (unnumbered, Department of Biological Sciences, North Carolina State University) of *C*. *mydas* (also naturally deceased) was collected from the New River, North Carolina, USA, in the fall of 2010 and has since been kept frozen. A naturally deceased hatchling loggerhead sea turtle, *Caretta caretta* (KPC16030906), was collected in the Kyoto Prefecture of Japan in August of 2010. Initially, KPC16030906 was fixed in 10% formaldehyde, but it has since been treated with 70% ethanol. A neonate leatherback sea turtle, *Dermochelys coriacea* (ZMUC R2106), stored in 70% ethanol, was acquired as a gift from ‘Danmarks Akvarium’ (former Danish Aquarium) in 1962, and has since been part of the vertebrate collection at the Zoological Museum, Natural History Museum of Denmark.

### Scanning electron microscopy (SEM)

Initial screening was performed using a Hitachi S-3400N SEM on uncoated fossil samples under low vacuum, and the elemental composition was determined via elemental mapping using EDX analysis (1,900 sec scanning time at 15 keV, 62.0 μA and a working distance of 10 mm). Following ToF-SIMS analysis, the samples were sputter-coated with a gold/palladium mixture and re-examined using a Zeiss Supra 40VP FEG-SEM (2 keV, working distance 3–5 mm, Everhart-Thornley secondary electron detector). Modern reference samples were sputter-coated with gold and analysed using a Tescan Mira3 High Resolution Schottky FEG-SEM (30 keV, working distance 3–5 mm, in-beam secondary electron detector).

### Transmission electron microscopy (TEM)

Both untreated and demineralised fossil matter was embedded in epoxy resin (AGAR 100 Resin kit, R1031), which was left to polymerise at room temperature for 72 hours, followed by 48 hours at 60 °C. Ultrathin sections (50 nm) of the infiltrated material were produced with a Leica EM UC7 Ultramicrotome using a diamond knife. The sections were then mounted to pioloform-coated copper grids.

Representative samples from the neck, carapace, plastron, and forelimbs of three extant sea turtle hatchlings (*Chelonia mydas*, *Caretta caretta* and *Dermochelys coriacea*) were collected using sterile scissors. The *C*. *mydas* and *C*. *caretta* samples were fixed overnight at 4 °C in 2.5% glutaraldehyde in 0.1 M phosphate buffer (pH 7.3). Following this treatment, the tissues were rinsed in 0.25 M sucrose in 0.1 M phosphate buffer (pH 7.3), and then fixed a second time for two hours at room temperature using 0.1 M buffer containing 1% osmium tetroxide. Thereafter, the integumental tissues were rinsed with a buffer solution and dehydrated in stepwise increased concentrations of ethanol. Finally, the tissues (together with *D*. *coriacea* skin dehydrated in a graded ethanol series) were embedded in epoxy resin (Quetol 651 and AGAR 100 Resin kit). Semithin sections (1.5 μm) were produced using a glass knife, whereas ultrathin sections (50 nm) were cut with a Leica Ultracut UC7 ultrotome using a diamond knife.

Ultrathin sections of all samples were examined in a JEOL JEM-1400 PLUS transmission electron microscope at 80 and 120 kV without further treatment or staining. Areas of interest were photographed using a JEOL Matataki CMOS camera.

### *In situ* immunohistochemistry

All manipulations of *Tasbacka* samples were conducted in a laboratory dedicated specifically to analyses of fossil materials, in which no modern tissues were handled or stored. All instruments, buffers and solutions were kept separated from those used in analyses of the modern controls. An aseptic protocol was applied; gloves, gowns and face masks were worn at all times. In a separate laboratory, a sterile scalpel was used to remove small pieces of muscle, claw and carapace scute material from a juvenile *Chelonia mydas*. The collected tissues were kept frozen at −80 °C in a freezer dedicated exclusively to modern samples, and in which no fossil material has ever been stored, until analysed.

Fossil samples or fixed *C*. *mydas* tissues (scute, claw and muscle) were dehydrated in 70% ethanol, then infiltrated with a 2:1 mixture of LR White (EMS Cat # 14383, hard grade) and 70% ethanol for one hour. Samples were then incubated, separately, in two changes of concentrated LR White for one hour, each time to equilibrate. All specimens were then embedded in concentrated LR White and allowed to polymerise at 60 °C for 24 hours. They were then sectioned to 200 nm using a Leica EM UC6 Ultramicrotome. Sections were collected and transferred to six-well, Teflon-coated slides, dried on a warming plate, then dried to completion in 45 °C oven overnight. Sections were etched with Proteinase K (PCR grade, Roche, 25 µg/ml) in ×1 phosphate buffered saline (PBS) buffer at 37 °C to expose epitopes, followed by two incubations in 500 mM EDTA (pH 8.0) and two incubations in 1 mg/ml sodium borohydride for 10 min each for epitope retrieval. All incubations were separated by sequential washes (two times for five min each) in PBS. Sections were then incubated for two hours with 4% normal goat serum (NGS) in PBS to occupy non-specific binding sites and prevent spurious binding. Sections were then incubated overnight at 4 °C in either primary antibody [Polyclonal Rabbit X-Alligator Haemoglobin (Biosynthesis BSYN6941) 1:100; Polyclonal Rabbit X-Ostrich Haemoglobin (GenScript 70594-1) 1:75; Polyclonal Rabbit X-Chicken Feather (Biosynthesis BSYN6734) 1:500; Polyclonal Rabbit X-Tropomyosin (Abcam ab11190) 1:50; or Monoclonal Mouse X-Peptidoglycan (AbD Serotec, 7263–1006) 1:75], diluted to final concentration in primary dilution buffer [(1% Bovine Serum Albumin (BSA) (Fisher, BP1660-100), 0.1% Cold Fish Skin Gelatine (Sigma G7765), 0.05% Sodium Azide (Sigma S-8032), 0.01 M PBS, pH 7.2], or in primary dilution buffer only, without primary antibody added to control for non-specific secondary antibody binding. All sections (test and control, fossil and modern) were washed thoroughly to remove unbound antibody, then incubated with secondary antibody [(Biotinylated Goat Anti-Rabbit IgG (H + L) (Vector BA-1000) diluted 1:500 for rabbit primary antibodies, Biotinylated Goat Anti-Mouse IgG (H + L) (Vector BA-9200), diluted 1:500 for monoclonal mouse anti-peptidoglycan] for two hours at room temperature. Fluorescein Avidin D (FITC, Vector Laboratories A-2001) diluted 1:1,000 was applied to all sections and allowed to bind for one hour at room temperature. All incubations were separated by sequential washes (two times for 10 min each) in PBS w/Tween 20 (ACROS Organics) followed by two 10 min rinses in PBS. Finally, the sections were mounted with Vectashield Anti-Fade mounting medium (Vector H-1000), and coverslips were applied. The sections were examined with a Zeiss Axioskop 2 Plus biological microscope and captured using an AxioCam MRc 5 (Zeiss) with ×10 ocular magnification; data were collected using the Axiovision software package (version 4.7.0.0).

The post-embedding TEM immunogold labelling protocol was applied to fossil soft tissue matter and, separately, to *C*. *mydas* scute material. Ultrathin (90 nm) sections were collected on carbon-coated nickel grids (EMS Cat # CFT200-NI). The sections were rinsed for 10 min by placing the grids on large droplets of PBS Tween-20. They were then incubated in 4% normal donkey serum (NDS) for one hour in PBS at room temperature to occupy non-specific binding sites and prevent spurious binding. Grids were incubated in primary antibody (Polyclonal Rabbit X-Chicken Feather; Biosynthesis BSYN6734) diluted to 1:20 in primary dilution buffer (as described above) for three hours at room temperature. The grids were washed on large droplets of PBS Tween-20 for 10 × 2 min, and then incubated with secondary antibody [12 nm Colloidal Gold AffiniPure Donkey Anti-Rabbit IgG (H + L) 1:20 (Jackson Immuno Research Inc Cat # 711-205-152)] for one hour. The grids were rinsed with PBS Tween-20 for 10 × 2 min, then in E-pure water for 3 × 30 sec, and finally dried with filter paper. The sections were observed using the Aberration Corrected STEM-FEI Titan 80–300 electron microscope at the Analytical Instrumentation Facility of North Carolina State University.

### Time-of-flight secondary ion mass spectrometry (ToF-SIMS)

ToF-SIMS is a surface analysis technique that provides spatially resolved molecular information from solid materials. During examination, a focussed beam of high-energy (primary) ions bombards the sample and mass spectra of the emitted (secondary) ions are recorded^66^. Ion images showing the spatial distribution of specific molecular species and mass spectra from selected structures are generated by scanning the primary ion beam across the sample surface.

In this study, both untreated and demineralised fossil samples were fixed for ToF-SIMS analysis on silicon wafers using either double-sided tape or Milli-Q water. Additionally, natural eumelanin from the cephalopod *Sepia officinalis* (Sigma-Aldrich) and hemin (MP Biomedicals) were fixed on solid substrates using double-sided tape. The fossil data were compared also to a number of other standard compounds, including various pheo- and pyomelanins^20–22^, hopanoids^67^, peptidoglycans (from *Micrococcus luteus* and *Methanobacterium* sp., provided by Sigma-Aldrich), and porphyrins [chlorophyll a^20^, protoporphyrin IX (Sigma-Aldrich), protoporphyrin X with copper (Sigma-Aldrich), uroporphyrin I dihydrochloride (Sigma-Aldrich), coproporphyrin I dihydrochloride^21^, and copper II phthalocyanine^21^].

ToF-SIMS analyses in the static SIMS mode were conducted in a TOFSIMS IV instrument (IONTOF GmbH) using 25 keV Bi_3_^+^ primary ions and low energy electron flooding for charge compensation. Positive and negative ion data were acquired with the instrument optimised either for high mass resolution (m/∆m ~5,000, spatial resolution ~3–4 µm) or high image resolution (m/∆m ~300, spatial resolution ~0.2–0.5 µm). The pulsed primary ion current was 0.10 pA for the high mass resolution data and 0.04 pA for the high image resolution data.

### Infrared (IR) microspectroscopy

Fossil tissues and sediments were removed from MHM-K2 using a sterile scalpel, suspended in Milli-Q water, and placed on a sterile CaF_2_ infrared window and left to air dry under a hood at room temperature. IR microspectroscopic measurements were recorded at beamline D7, MAX-IV laboratory, Sweden. The set-up combines a Hyperion 3000 microscope and a Bruker IFS66/v FTIR spectrometer. The infrared microscope was operated in transmission mode using a 170 × 170 µm aperture, a single element MCT, a 100 × 100 µm detector, and a ×15 objective/condenser. This arrangement gives a visible magnification of ×215 for the video camera in the microscope, which was used to locate relevant structures in the sample.

Measurements were taken also at the Department of Biology, Lund University. Here, a Hyperion 3000 microscope combined with a Tensor 27 spectrometer was used together with a single element MCT detector (250 × 250 µm) and a Globar light source. The microscope was operated in transmission mode at 4 cm^−1^ resolution, and a ×15 objective was employed. 128 scans were averaged to give a good signal to noise ratio.

### Data availability

The datasets generated during the current study are available from the corresponding author on reasonable request.

## Electronic supplementary material

Supplementary information
