# Supplementary material for: Biochemistry and adaptive colouration of an exceptionally preserved juvenile fossil sea turtle
Source: Sci Rep. 2017 Oct 17;7:13324. doi: 10.1038/s41598-017-13187-5 (PMC5645316; doi:10.1038/s41598-017-13187-5)
Supplement: Supplementary file 1 — Supplementary information [file 41598_2017_13187_MOESM1_ESM.doc]

**SUPPLEMENTARY INFORMATION FOR**

**Biochemistry and adaptive colouration of an exceptionally preserved juvenile fossil sea turtle**

Johan Lindgren^1,*^, Takeo Kuriyama^1,2,3^, Henrik Madsen^4^, Peter Sjövall^5^, Wenxia Zheng^6,7^, Per Uvdal^8^, Anders Engdahl^9^, Alison E. Moyer^6^, Johan A. Gren^1^, Naoki Kamezaki^10^, Shintaro Ueno^11^ & Mary H. Schweitzer^1,6,7^

*^1^Department of Geology, Lund University, 223 62 Lund, Sweden. ^2^Institute of Natural and Environmental Sciences, University of Hyogo, 669 3842 Hyogo, Japan. ^3^Wildlife Management Research Center, 669 3842 Hyogo, Japan. ^4^Mo-clay Museum, 7900 Nykøbing Mors, Denmark. ^5^RISE Research Institutes of Sweden, Chemistry and Materials, 501 15 Borås, Sweden. ^6^Department of Biological Sciences, North Carolina State University, Raleigh, NC 27695, USA. ^7^North Carolina Museum of Natural Sciences, Raleigh, NC 27601, USA. ^8^Chemical Physics, Department of Chemistry, Lund University, 221 00 Lund, Sweden. ^9^MAX-IV laboratory, Lund University, 221 00 Lund, Sweden. ^10^Department of Biosphere-Geosphere Science, Okayama University of Science, 700 005 Okayama, Japan. ^11^Department of Ecosystem Studies, University of Tokyo, 113 8657 Tokyo, Japan.*

*^*^Correspondence and requests for materials should be addressed to J.L. (email:* [*johan.lindgren@geol.lu.se*](mailto:johan.lindgren@geol.lu.se)*)*

**
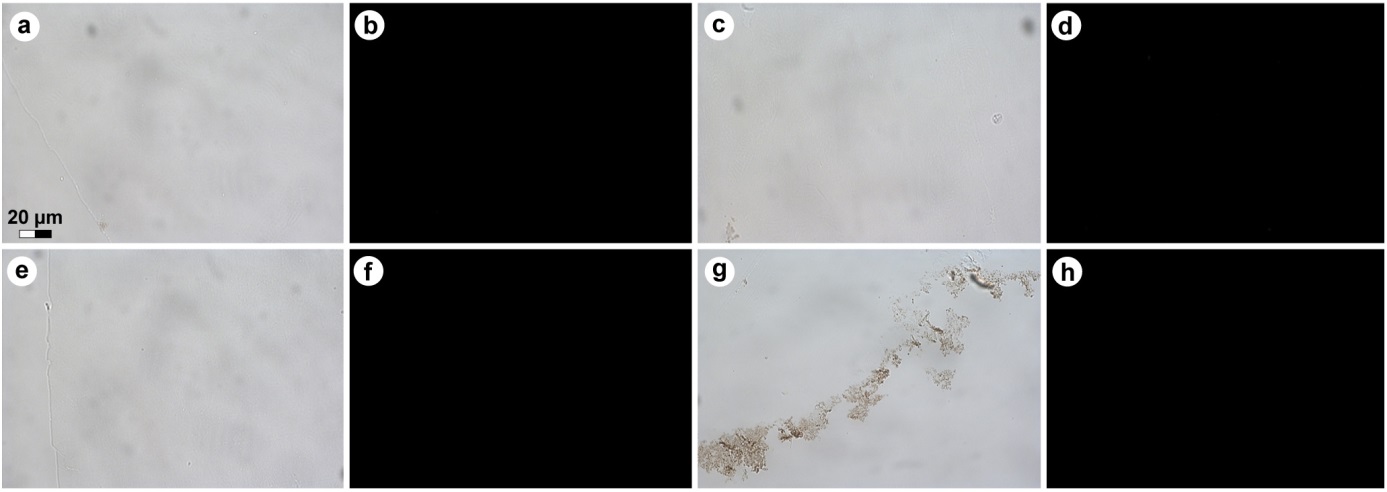
**

**Supplementary Figure S1. Representative negative controls used in the immunohistochemical analyses.** (**a**, **b**) No primary antibody applied to extant *Chelonia mydas* muscle tissue to control for spurious binding of secondary antibody and/or fluorescent label. (**c**, **d**) To control for non-specific binding of the primary antibodies, *C*. *mydas* muscle tissue was exposed to antibodies raised against modern chicken feather. Because muscles are not found in association with keratinous proteins, no reactivity was expected or observed. Note that antibodies to haemoglobin and tropomyosin (both associated with muscles) did bind to this tissue (Fig. 3g, h, k, l, o, p). (**e**, **f**) Similarly, *C. mydas* claw sheath material was exposed to tropomyosin antibodies without apparent binding, supporting the specificity of these antibodies. (**g**, **h**) *Tasbacka danica* soft tissue matter exposed to human elastin. No reactivity was expected or observed. **a**, **c**, **e**, **g** are overlay images, superimposing fluorescent signal on transmitted light image of sectioned tissue to reveal the localisation of antibody-antigen complexes to tissue. **b**, **d**, **f**, **h** are imaged using a FITC filter.

**
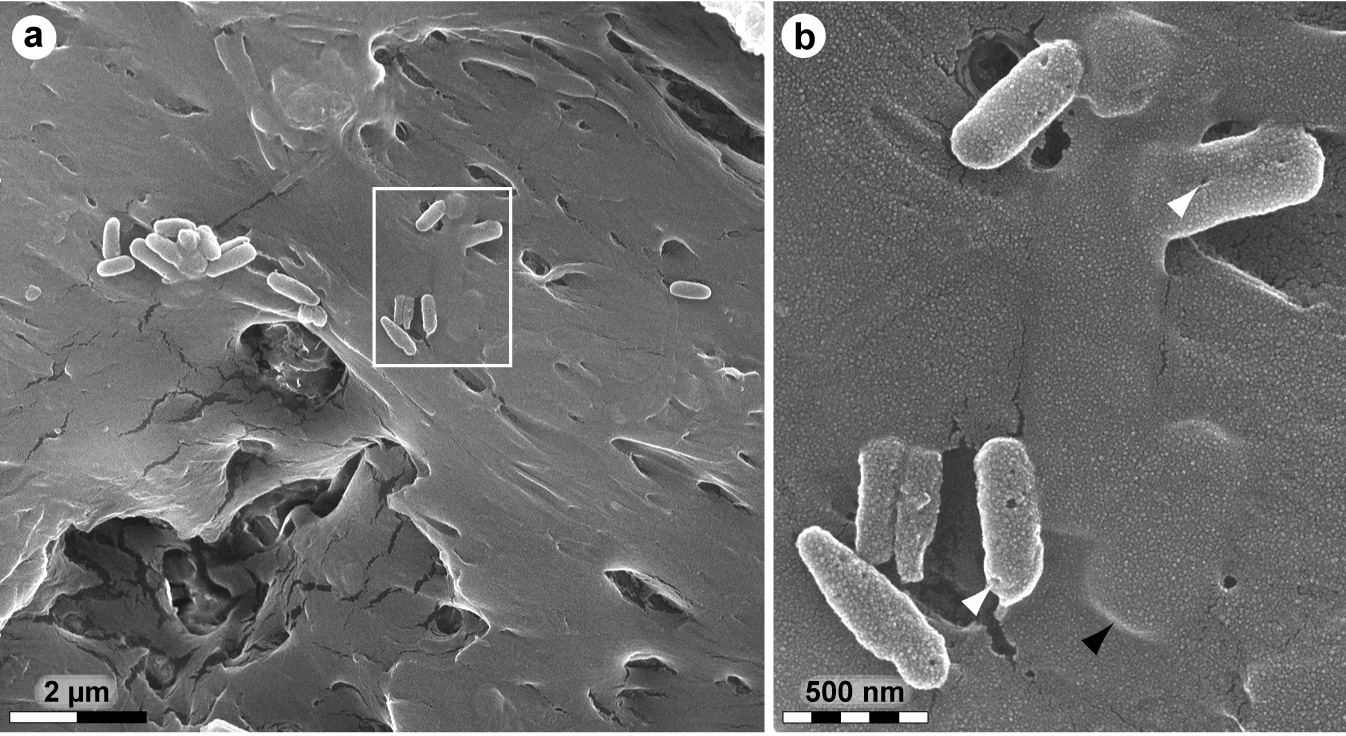
**

**Supplementary Figure S2. FEG-SEM fractographs of the pigmented keratinous epidermal coating** **of a hatchling loggerhead sea turtle, *Caretta caretta* (KPC16030906).** (**a**) Tangential view of the carapace keratin laminate depicting layers of cornified proteins and clusters of elongate melanosomes (see also Supplementary Fig. S4g, h). (**b**) Demarcated area in **a** showing melanosome organelles tightly adhering to, partially embedded in, and even fully enclosed by (black arrowhead) the keratinous substrate. White arrowheads indicate scattered pits on the surface of the melanosomes.

**
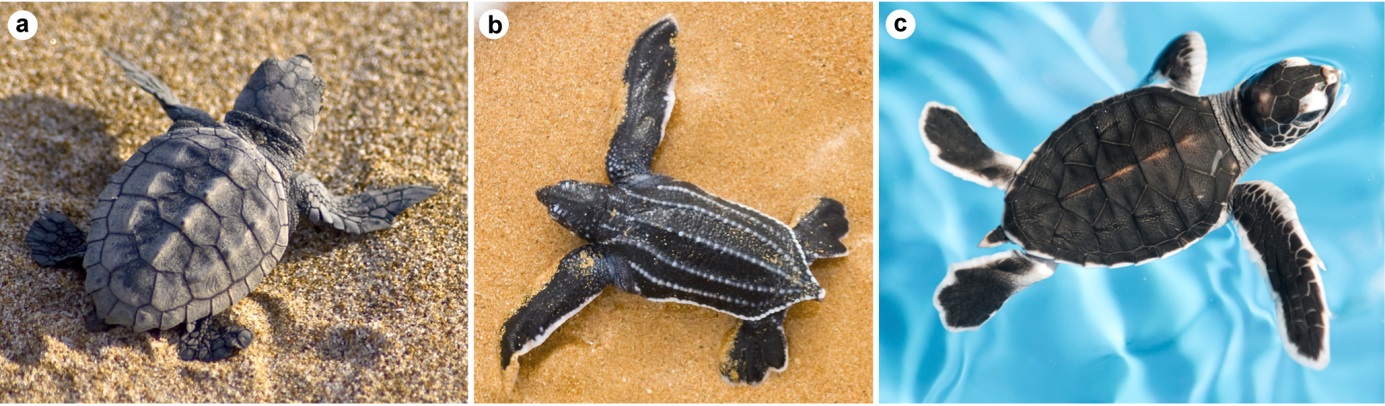
**

**Supplementary Figure S3. Dorsal colour patterns of three extant sea turtle hatchlings (images are not to scale).** (**a**) Loggerhead sea turtle, *Caretta caretta*. Photo curtesy of [Benjamin Albiach Galan](http://www.shutterstock.com/gallery-251074p1.html)/Shutterstock.com. (**b**)Leatherback sea turtle, *Dermochelys coriacea*. Photo curtesy of [IrinaK](http://www.shutterstock.com/gallery-117169p1.html)/Shutterstock.com. (**c**)Green sea turtle, *Chelonia mydas*. Photo curtesy of [Kim Pin](http://www.shutterstock.com/gallery-3027p1.html)/Shutterstock.com.

**
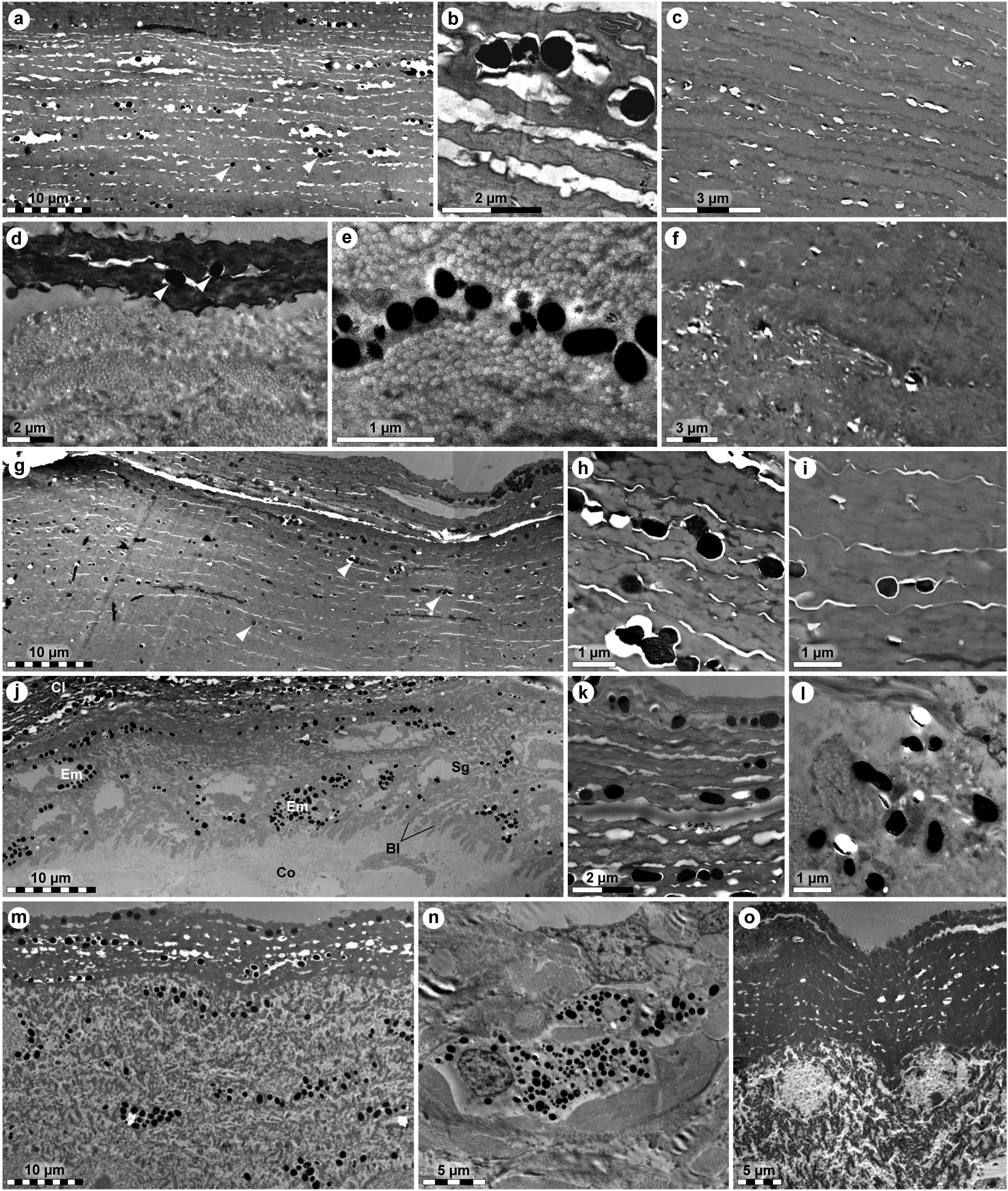
**

**Supplementary Figure S4. TEM micrographs of integumental tissues obtained from (a–f) *Chelonia mydas*, (g–l) *Caretta caretta* and (m–o) *Dermochelys coriacea*.** (**a**) Vertical section through the darkly pigmented keratinous epidermal coating of the carapace of a hatchling green sea turtle, *C*. *mydas* (KPC16030901). Arrowheads indicate melanosomes (note electron-dense interior and intimate association with the fibrous matrix). (**b**) Higher magnification micrograph of the carapace keratin laminate in KPC16030901 depicting melanosomes embedded in cornified proteins. (**c**) Vertical section through a light-coloured portion of the keratinous epidermal coating of the plastron in KPC16030901. Note absence of pigment organelles. (**d**) Detail of the epidermis of dark-coloured flipper skin in KPC16030901 showing melanosomes (arrowheads). (**e**) Detail of the dermis of dark-coloured flipper skin in KPC16030901 depicting a melanophore dendrite. (**f**) Vertical section through light-coloured ventral forelimb epidermis in KPC16030901. Note absence of melanosomes. (**g**) Vertical section through the pigmented keratinous epidermal coating of the carapace of a hatchling loggerhead sea turtle, *C*. *caretta* (KPC16030906). Arrowheads indicate melanosomes. (**h**) Higher magnification micrograph of the keratin laminate in KPC16030906 depicting melanosomes embedded in cornified proteins. (**i**) Vertical section through the dark-coloured keratinous epidermal coating of the plastron in KPC16030906. Note presence of melanosomes. (**j**) Vertical section through dark-coloured flipper skin in KPC16030906. Bl, basal lamina; Cl, cornified layers; Co, collagen fibres; Em, epidermal melanophore; Sg, stratum germinativum. (**k**) Detail of the epidermis of dark-coloured flipper skin in KPC16030906 showing abundant melanosomes. (**l**) Dermal melanophore of dark-coloured flipper skin in KPC16030906. (**m**) Vertical section through the dark-coloured epidermis of the carapace of a neonate leatherback sea turtle, *D*. *coriacea* (ZMUC R2106). (**n**) Higher magnification micrograph of a dermal melanophore of black-coloured flipper skin in ZMUC R2106. (**o**) White-coloured epidermis from the trailing edge of a flipper in ZMUC R2106. Note absence of melanosomes.
